# Evaluation of kidney disease using e-GFR compared to measured creatinine clearance

**DOI:** 10.6026/973206300200229

**Published:** 2024-03-31

**Authors:** SP Tejaswi Pullakanam, Barla Krishna, Nakka Madhuri, Priya K Dhas, Nekkala Ramakrishna

**Affiliations:** 1 Department of Biochemistry, Gayatri Vidya Parishad Institute of Health Care and Medical Technology, Marikavalasa, Visakhapatnam, Andhra Pradesh, India; 2 Department of Biochemistry, Vinayaka Missions Kirupananda Variyar Medical College and Hospitals, Salem, Tamilnadu, India

**Keywords:** Serum creatinine (SCr), creatinine clearance (CrCl), glomerular filtration rate (GFR), Cockcroft-Gault formula (CG), chronic kidney disease (CKD)

## Abstract

Measurement of renal function is required for diagnosis and stratification of kidney disease. GFR is considered as the best overall measure of kidney function for diagnosis and treatment of patients with CKD. Measuring GFR is time consuming and hence eGFR is calculated using equations with endogenous markers like SCr. Therefore, it is of interest to examine the accuracy of creatinine based estimates (CrCl and CG formula) of GFR among patients. Thus, 60 in-patients (30 men and 30 women) at the GVP hospital and 40 controls were enrolled in the study. SCr and 24 hrs urine creatinine are estimated using blood sample and same day 24-hr urine collection. SCr is estimated using the Kinetic Jaffe's method in Auto analyzer for serum and urine. eGFR is calculated using the CG formula for the SCr value. We evaluated the correlation between measured CrCl derived from 24-hr urine collection and calculated/predicted CrCl using the CG equations. A positive correlation was observed between measured GFR and e-GFR in case and control groups.

## Background:

Chronic kidney disease (CKD) is a progressive disease involving the irreversible loss of kidney function over the time [1]. Under normal physiologic conditions, the kidneys serve several functions: such as-regulation of fluid volume, acid base balance of plasma, excretion of nitrogenous waste, synthesis of erythropoietin, 1,25-dihydroxy-cholecalciferol, and renin, and different drug metabolism. However, due to progression of renal dysfunction CKD is associated with multiple physiological and metabolic disturbances, such as-worsening and eventual failure of kidney function, accumulation of uremic toxins, metabolic acidosis, abnormalities in lipid, amino acid, mineral metabolism, malnutrition, insulin resistance, inflammation, oxidative stress, anemia, vitamin D deficiency, skeletal muscle dysfunction, hypertension, and anorexia-Cachexia which are linked to poor outcomes [2]. Chronic Kidney Disease (CKD) is defined as kidney damage of three or more months duration caused by structural or functional abnormalities with or without a decreased glomerular filtration rate (GFR). Pathological markers, abnormalities in the blood or urine, or imaging tests, may reveal kidney dysfunction [3]. CKD was defined as creatinine clearance (CrCl) or GFR less than 60 ml/min/1.73 m2 [4, 5] here are, three equations in use to estimate eGFR: Four-variable MDRD equation [6, 7] CKD-EPI equation [8] and Cockcroft-Gault equation [9]. The KDIGO recommends that CKD be diagnosed, classified, and staged by GFR [10]. In clinical practice GFR is crucial for diagnosis, management, drug dosing and prognosis, in addition to its utility for research and public health [11, 12 & 13]. The national kidney foundation kidney disease outcomes quality initiative (NKF-K/DOQI) framed a classification system that has become widely adopted in clinical practice (Table 1). Different degrees of renal dysfunction [1] from the earliest kidney damage to end-stage renal disease (ESRD) have been classified into five stages on the basis of level of kidney function based on (glomerular filtration rate) [1]. CKD classification is relevant as it has been associated with outcomes such as kidney disease progression, cardiovascular disease and all-cause mortality. It is also important as it can allow therapeutic interventions in earlier stages to slow disease progression reduce complications related to decreased estimated GFR (eGFR), cardiovascular (CVD) risk and improve quality of life and survival (Table 1) [2,3, 4]. Glomerular filtration rate is measured (mGFR) indirectly as the clearance of filtration markers that are eliminated by the kidney only by glomerular filtration. Clearance can be measured as either plasma or urinary methods that record the clearance of endogenous or exogenous substances by the kidney [11]. Creatinine: Serum creatinine is the most commonly used endogenous glomerular filtration marker in clinical practice [14, 15 & 16]. Therefore, it is of interest to determine the utility of eGFR in comparison to measured creatinine clearance in the evaluation of kidney disease.

## Materials and Methods:

## Materials:

The Present study was conducted at the Department of Biochemistry, Gayatri Vidya Parishad Hospital and Medical College (GVPIHC&MT) Visakhapatnam, Andhra Pradesh, India. Written informed consent was obtained from all subjects enrolled. The study was approved by the Institutional Ethical Committee (IEC).

## Subjects:

Patients attending the Department of Nephrology, Gayatri Vidya Parishad Hospital, with established diagnosis of CKD were subjects of the study. A total of (n=60) CKD patients were included in the study. Patients were classified into various stages of CKD (30 men and 30 women) based on GFR (calculated from serum creatinine, using cockcroft-gault (CG) equation. Healthy volunteers (n=40) who were enrolled in this study served as controls.

## Inclusion criteria:

Patients with adult age, Patients with stage I-V CKD

## Exclusion criteria:

Pediatric patients, patients undergoing either hemo-dialysis or peritoneal dialysis treatment, organ transplantation & other active or chronic infections, patients taking anti-inflammatory and immunosuppressive drugs were excluded.

## Sample Collection:

Two micro litre of blood was drawn and transferred into a plain tube . collected from all the participants. Samples were separated by centrifugation and stored at -50°C until further biochemical analysis.

## Estimation of serum creatinine:

was analyzed by using Modified Jaffe's (UV-Kinetic) [17].

## Statistical analysis:

Data were entered in MS-Excel and analyzed in SPSS V25. Descriptive statistics were represented with percentages, Mean with SD or Median with IQR depends on nature of the data. Shapiro wilk test was applied to find normality. Chi-square test, Mann-whitney U test, Spearman correlation were applied. P<0.05 was considered as statistically significant.

## GFR Cockcroft - Gault Equation:

Mg of substance excreted per minute in urine clearance = Mg of substance per ml of plasma

C = U x V/P =ml/mt

## Results:

The renal images of CKD patients from whom the samples were collected and analyzed (data not shown - check with authors). Table 2 shows that maximum and minimum age group ranges in cases were 32-80 years and in controls were 22-29 years range. The mean age ± SD in cases was 56.23 ± 10. 44 and in controls was 24.98 ± 1. 88. Table 3 shows that among a total of 60 cases & 40 controls, 50% of cases are females and remaining 50% of cases are males. Out of 40, 50% of controls are females and remaining 50% of are males. Equal distribution among cases and controls was maintained. Table 4 shows that the maximum weight in group cases is 78 kgs and controls are 75 kgs. Weight (Mean ± SD) in case group is 60.68 ± 6.90 and in the control group the weight (Mean ± SD) is 58.90 ± 6.48. Table 5 shows that maximum range of serum creatinine in cases is 9.65 mg/dl and controls are 0.60 mg/dl. Mean ± SD of serum creatinine in case group is 5.82 ± 1.29 and in control group Mean ± SD is 0. 87±0. 16. Table 6 shows that maximum range of urinary creatinine in cases is 89. 00mg/dl and controls are 97. 60 mg/dl. Mean ± SD of urinary creatinine in case group is 62. 58 ± 15.99) and in control group is mean ± SD is 97.60 ± 12.67. Table 7 shows that maximum range of urinary volume in cases is 3000ml and controls are 2300 (ml). Mean ± SD of urine volume in case group (3000 ± 491. 16), whereas in control group, Mean ± SD (2300 ± 228.69). Table 8 shows that maximum range of eGFR in cases is 27. 26 (ml/min/1. 73 m2) and controls is 136.96 (ml/min/1. 73 m2). Mean ± SD of eGFR in case group is 27.26 ± 3.75) and in control group mean ± SD is 136.96 ± 11.37. Table 9 shows that maximum range of CG in cases is 28.16 (ml/min/1. 73 m2) and controls is 127.78 (ml/min/1. 73 m2). Mean ± SD of CG in case group is 28.16 ± 3.86 and in control group mean ± SD is 127.78 ± 16.42. Table 10 shows the positive and significant correlation between the variables of both control and cases.

## Discussion:

Chronic Kidney Disease (CKD) is defined as kidney damage caused by structural or functional abnormalities with or without a decreased glomerular filtration rate (GFR). There are various methods available for the estimation of eGFR which is measured using the GFR Cockcroft - Gault equation that is used in the estimation of eGFR. Measured GFR includes the procedure of 24-hour urine collection by estimating the serum creatinine and urinary creatinine values. We included 60 persons (men & women) having chronic kidney disease and 40 healthy volunteers (men & women) in this study. Out of 60 subjects, the mean age is 56.2 ± 10.44 with a range of 32-80 years for subjects and it is 24.9 ± 1.88 for controls. Studies from other parts of India reported that 75% of the study subjects are in the age group of 65 years or older as shown by Liu *et al.* [18]. Current data shows that the maximum value of serum creatinine in the case group is 9.65 mg/dl and the minimum value observed in the case group is 2.90 mg/dl. Qiu *et al.* [19] showed that for the assessment of renal function, the cut - off value of SCr (serum creatinine) is 0.85.-1.69.0 mg/dl. Among 60 subjects, the mean ± SD of urinary creatinine was (62.58 ± 15.99) in case group and whereas in control group, the mean ± SD (73.76± 12.67). The urinary creatinine is similar with Lucia *et al.* [20] and it assessed that lower urine creatinine excretion predicts greater risk of kidney failure and patient mortality. This is also similar to Kumar *et al.* [21]. Data shows that among 60 subjects, the maximum range of urinary volume in cases is 3000 ml and controls is 2300ml. Mean ± SD of urine volume in case group is 3000 ±491.16 and whereas in control group the mean ± SD is 2300 ± 228.69. This is in accordance with Xu *et al.* [22]. Data shows that among 60 subjects the maximum range of eGFR in cases is 27.26 (ml/min/1.73 m2) and controls is 136.96 (ml/min/1.73 m2). The mean ± SD of eGFR in case group is 27.26 ± 3.75 and in control group, the mean ± SD is 136.96 ± 11.37. Further, among 60 subjects shows that maximum range of CGin cases is 28.16 (ml/min/1.73 m2) and controls is 127.78 (ml/min/1.73 m2). Mean ± SD of CG in case group is 28.16±3.86) and in control group, the mean ± SD is 127.78±16.42. This is in accordance with Kumar *et al.* [21] that showed the creatinine based GFR estimation provides a more accurate assessment of 24 hour creatinine clearance and kidney function. Thus, the use of Cockcroft-Gault formulae in estimating GFR for drug dosing, detection of CKD and for prognosis is evident. Further, a positive linear correlation between mGFR & eGFR and Cockcroft gaunt in controls and CKD patients in accordance with Kumar *et al.* [21] is observed.

## Conclusion:

Data shows that e-GFR obtained by cockcroft gault using creatinine value is having good correlation with measured GFR using serum creatinine and 24 hrs urine creatinine profile. Thus, e-GFR is useful for the evaluation of kidney disease in hospital setting.

## Support:

Nil

## Figures and Tables

**Table 1:** The 5 Stages of CKD defined by Estimated Glomerular Filtration Rate [1]

**Stage**	**Description**	**GFR Action (ml/min/1.73 m2)**	**Action**
1	Kidney damage with, normal or ↑ GFR	>90	Diagnosis and Treatment of comorbid conditions, Slowing Progression, CVD risk reduction
2	Kidney damage with mild↓GFR	60-89	Estimating Progression
3	Moderate ↓ GFR	30-59	Evaluating &treating complication
4	Severe ↓ GFR	15-29	Preparation for RRT
5	Kidney failure	<15	Renal Replacement Therapy(RRT)

**Table 2:** Comparison of the Variable AGE (in Years) in Controls and CKD patients

**Variable**	**Group**	**Minimum**	**Maximum**	**Mean**	**SD**	**Median**	**IQR**	**P-value**
Age	Case	32	80	56.23	10.44	56.5	17	<0.00
	Control	22	29	24.98	1.83	24.5	2	
SD-Standard deviation,
IQR-Interquartile range,
(S): Significant (p<0.001) P-value means the probability of obtaining results at least as extreme as the observed results of a statistical hypothesis test, assuming that the null hypothesis is correct.

**Table 3:** Distribution of Gender (Female & Male) in controls and CKD patients

**Sex**	**Case**		**Control**	
	**Count**	**%**	**Count**	**%**
Female	30	50.00%	20	50.00%
Male	30	50.00%	20	50.00%
Total	60	100.00%	40	100.00%
P-value=1				

**Table 4:** Comparison of variable (weight) in controls and CKD patients

**Variable**	**Group**	**Minimum**	**Maximum**	**Mean**	**SD**	**Median**	**IQR**	**P-value**
Weight	Case	46	78	60.68	6.9	60	10	0.253
	Control	45	75	58.9	6.48	59	8.5	

**Table 5:** Comparison of the variable serum creatinine (in mg/dl) in controls and CKD patients

**Variable**	**Group**	**Minimum**	**Maximum**	**Mean**	**SD**	**Median**	**IQR**	**P-value**
SCr (mg/dl)	Case	2.9	9.65	5.82	1.29	5.6	2	<0.001
	Control	0.6	1.2	0.87	0.16	0.9	0.2	

**Table 6:** Comparison of the variable urine creatinine (mg/dl) in CKD patients

**Variable**	**Group**	**Minimum**	**Maximum**	**Mean**	**SD**	**Median**	**IQR**	**P-value**
Urinary Creatinine (mg/dl)	Case	28	89	62.58	15.99	65	21.75	<0.001
	Control	40.6	97.6	73.76	12.67	75.55	17.73	

**Table 7:** Comparison of the variable urine volume (in ml/24hrs) CKD patients & controls

**Variable**	**Group**	**Minimum**	**Maximum**	**Mean**	**SD**	**Median**	**IQR**	**P-value**
Urine volume (ml)	Case	960	3000	1525.33	491.16	1385	537.5	<0.001
	Control	1445	2300	1849.25	228.69	1855	332.5	

**Table 8:** Comparison of the variable eGFR (ml/mt) in controls and CKD patients

**Variable**	**Group**	**Minimum**	**Maximum**	**Mean**	**SD**	**Median**	**IQR**	**P-value**
eGFR (ml/min/1.73 m2)	Case	6.6	27.26	11.25	3.75	10.38	4.8	<0.001
	Control	92.08	136.96	116.15	11.37	118.39	18.97	

**Table 9:** Comparison of the variable (Cockcroft -Gault) in controls and CKD patients

**Variable**	**Group**	**Minimum**	**Maximum**	**Mean**	**SD**	**Median**	**IQR**	**P-value**
CG ml/min/1.73 m2)	Case	6.29	28.16	11.83	3.86	11.27	5.19	<0.001
	Control	69.81	127.78	102.05	16.42	102.8	28.64	

**Table 10:** Correlation between variables of (eGFR and Cockcroft Gault) in (controls and CKD patients)

**Group**	**Correlation between eGFR (ml/min/1.73 m2) & CG ml/min/1.73 m2)**	**P-value**
Case	.947**	<0.001
Control	.522**	<0.001
